# A Backwards Approach to GD2 Immunofluorescence in Human Neuroblastoma Tissue Samples: From Staining to Slicing

**DOI:** 10.3390/cells14181462

**Published:** 2025-09-18

**Authors:** Sara Peggion, Clara Volz, Magdalena Trochimiuk, Isabelle Ariane Bley, Júlia Ramos, Konrad Reinshagen, Laia Pagerols Raluy

**Affiliations:** 1 Department of Pediatric Surgery, University Medical Centre Hamburg-Eppendorf, Martinistrasse 52, 20246 Hamburg, Germanyk.reinshagen@uke.de (K.R.); 2 Division of Pediatric Stem Cell Transplantation and Immunology, Clinic of Pediatric Hematology and Oncology, University Medical Center Hamburg-Eppendorf, 20246 Hamburg, Germany; 3 Research Institute Children’s Cancer Center Hamburg, 20251 Hamburg, Germany; 4 Institut Bonanova, Circumval.lació 8, 08003 Barcelona, Spain

**Keywords:** disialoganglioside, GD2, neuroblastoma, immunofluorescence staining

## Abstract

Background: The disialoganglioside GD2, located at the plasma membrane, is selectively overexpressed in various solid tumors, where it contributes to tumor growth and the development of an aggressive tumor phenotype. Thus, over the last two decades GD2 has been gaining importance both as a tumor marker and a therapy target. In neuroblastoma, anti-GD2 monoclonal antibodies and CAR T-cells have become an integral part of the multimodal treatment for relapsed or refractory high-risk cases, which continue to associate with poor prognosis. GD2 characterization in neuroblastoma is well established for bone marrow aspirates and biopsies, but remains challenging in tumoral tissue samples, mostly due to epitope loss upon fixation. Aims: The aim of our work was to assess a new protocol by staining GD2 in tissue specimens prior to fixation. Methods: Positive controls were tissue specimens from patients with histologically confirmed neuroblastoma and GD2 expression in bone marrow aspirate (*n* = 5). Nephroblastoma or Hodgkin lymphoma samples were considered as negative controls (*n* = 5). Tissue staining was performed prior to fixation with either anti-GD2 antibody or isotype control, followed by secondary antibody staining and subsequent paraffinization. To examine GD2 staining before and after paraffinization, fluorescence images were acquired using 3D and 2D immunofluorescence microscopy techniques respectively. Results: GD2 signal was detected in all positive controls, while absent in all negative controls. After fixation, paraffinization and slicing no relevant signal loss was observed. Nevertheless, sufficient staining of 3D specimens required long incubation times, which led to increased cytolysis of the unfixed tissue. Conclusions: We were able to establish and validate a novel protocol to reliably perform immunostaining of the membrane antigen GD2 in unfixed, primary neuroblastoma tissue. Although including few limitations, this staining workflow enables relatively quick assessment of GD2 status and thus, might represent a relevant diagnostic tool within the framework of tumor staging and precision medicine.

## 1. Introduction

Gangliosides are amphiphilic molecules, which represent a sialic acid-containing subgroup of glycosphingolipids; a hydrophobic ceramide links covalently to a hydrophilic oligosaccharide. They are mainly located on the outer layer of neuronal membranes, where they form microdomains exerting immunogenic functions [1,2]. Their anomalous expression characterizes several tumors. Specifically, the disialoganglioside GD2 fosters cell survival, tumor invasion and enhances tumoral immune escape mechanisms. It is highly expressed in tumor entities of neuroectodermal origin [3]; among them, neuroblastoma (NB) is the most common solid extracranial tumor affecting pediatric patients, who are classified into subsets according to their risk of death. Although patients with high-risk disease (about 60%) still present a 5-year overall survival rate of approximately 50% despite multimodal therapeutic regimens, the introduction of GD2-targeted immunotherapy in treatment protocols has improved their outcome [4]. Currently, monoclonal antibodies (mAb) targeting GD2 (e.g., dinutuximab) are the standard of care for high-risk NB patients [5]. More recently, the use of chimeric antigen receptor-expressing T cells targeting GD2 (GD2 CAR T-cells) was demonstrated to be feasible and safe for treating relapsed or refractory high-risk NB [6].

Since NB is deemed as GD2-positive, GD2 expression analysis is not necessary prior to anti-GD2 immunotherapy [3,6]. GD2 immunocytology alone is well established for the quantitative analysis of NB contamination in bone marrow aspirates and biopsies [7]. However, the analysis of ganglioside expression is particularly difficult with standard methodologies, such as immunohistochemistry on formalin-fixed, paraffin-embedded (FFPE) samples, since ceramides are dissolved from the tissue in the process [3].

Nevertheless, the abovementioned immunotherapeutic strategies have shown several pitfalls; e.g., mAb are not effective in cases of low or heterogenous antigen expression [6]. Furthermore, although rare, multiple cases of lost or lacking immunocytological GD2 expression in NB cells from bone marrow samples have been reported [8,9]. To the best of our knowledge, this aspect has not been fully addressed in NB tumor tissue samples yet, although it might have significant diagnostic and therapeutic consequences. Determining levels of GD2 expression could help to stratify patients receiving anti-GD2 therapies.

In the present work, we established a novel protocol to perform GD2 immunofluorescence (IF) staining in unprocessed tumor tissue samples, with a focus on neuroblastoma. We also checked for possible epitope loss, when stained specimens undergo standard processing (formalin fixation and paraffin embedding). 

Our results confirm the feasibility and reproducibility of the protocol; the staining allows reliable and specific detection of GD2 in neuroblastoma. The acquired signal is detectable both in unfixed stained tissue as well as in FFPE-slices, which increases the applicability of this new technique. 

## 2. Materials and Methods

### 2.1. Source of Samples

Tumor tissue samples were obtained by biopsy at initial patient presentation or by surgery after neoadjuvant chemotherapy. For method establishment and validation, patients with a histologically confirmed diagnosis of neuroblastoma and GD2-positive expression in bone marrow aspirate were enrolled as positive controls (n = 5). Samples derived from patients with histologically confirmed nephroblastoma or Hodgkin lymphoma were considered negative controls (n = 5), since both entities are not known to overexpress GD2 [10,11,12].

Either fresh tissue (preserved in NaCl 0.9% solution upon collection) or thawed frozen tissue can be stained by using the following protocol. Tissue cryopreservation (in the gas phase of liquid nitrogen) should be performed using a freezing medium (e.g., Recovery Cell Culture Freezing Medium, Gibco-Cat. No. 12648-10, Thermo Fisher Scientific, Grand Island, New York, USA) for optimal tissue characteristic maintenance. Multiple freeze–thaw cycles should be avoided, based on our experience.

Specimens obtained from the samples for staining according to the following protocol should not exceed a maximum thickness of 3 mm.

### 2.2. Antibodies

Tissue specimens were stained with either Purified Mouse anti-Human Disialoganglioside GD2–Clone 14.G2a (BD Pharmingen™, Cat. No. 554272, Becton, Dickinson and Company, San Diego, California, USA) or a Purified Mouse IgG2a, κ Isotype Control (BD Pharmingen™, Cat. No. 556651, Becton, Dickinson and Company, San Diego, CA, USA). For the establishment of proper primary antibody conditions, two different working concentrations were tested: tissue specimens were incubated with both abovementioned antibodies with a concentration of either 2.5 µg/mL or 1 µg/mL for either 2 or 4 hours at room temperature (RT).

A Donkey Anti-Mouse IgG H&L conjugated with the fluorochrome Alexa Fluor^®^ 647 (abcam, Cat. No. ab150107, Cambridge, UK) was used as a secondary antibody at a final concentration of 10 µg/mL for 90 minutes at RT, according to the manufacturer’s reactivity data.

Additionally, antibody-mediated background signal, antibody specificity, and absence of autofluorescence were tested by incubating the abovementioned samples with either primary or secondary antibody alone, or with distilled water.

### 2.3. Antibody Validation Strategy

Before protocol development, the antibody’s selective affinity for GD2 was tested on NB cell lines. LS and LS KO-B4GALNT1 were kindly provided by Prof. Dr. Ingo Müller (Section for Pediatric Stem Cell Transplantation and Immunology, University Medical Center Hamburg-Eppendorf, Hamburg-Eppendorf, Hamburg, Germany). GD2 expression is well documented in LS cells [13], whereas the expression of the gene encoding the enzyme beta-1,4-N-Acetylgalactosaminyltransferase 1 (GM2/GD2 synthase) is knocked out (KO) in LS KO-B4GALNT1 cells. Since this is a key enzyme that catalyzes the conversion of GD3 to GD2, its knock out via CRISPR/Cas9 leads to a complete loss of GD2 synthesis [14].

The abovementioned cells were initially cultured in T-175 flasks at 37 °C in a 5% CO_2_ incubator (Panasonic Healthcare Co., Ltd., Model No. MCO-19AICUV-PE, Tokyo, Japan) with 25 mL RPMI-1640 (1×) Medium (Gibco, Cat. No. 42401-018, Life Technologies^TM^ Ltd., Paisley, Scotland, UK) supplemented with 10% fetal bovine serum (FBS) (Gibco, Cat. No. 26140079, Thermo Fischer Scientific, Waltham, Massachusetts, USA), 2 mM L-glutamine (Thermo Fischer Scientific, Cat. No. 25030081, Carlsbad, CA, USA) and 100 µg/mL Normocin™ (InvivoGen, Cat. No. ant-nr-2, San Diego, CA, USA).

By a confluence of about 70%, cells were treated with Accutase® (CAPRICORN, Cat. No. ACC-1B, Gleichen, Lower Saxony, Germany) over 5 minutes at 37 °C in a 5% CO_2_ incubator and seeded on glass coverslips in a 12-well assay plate in a density of 1 × 10^6^ cells/well. Twenty-four hours later, unfixed cells were washed with Dulbecco’s Phosphate-Buffered Saline (DPBS) (Gibco, Cat. No. 14190-094, Grand Island, Nebraska, USA) twice for five minutes and blocked for 60 minutes with DPBS containing 2% bovine serum albumin (BSA) (Sigma Aldrich, Cat. No. A9418, St. Louis, MI, USA) and 1% FBS. Subsequently, cells were incubated with either primary anti-GD2 or isotype control antibodies in DPBS (2.5 µg/mL) for 90 minutes at room temperature (RT).

After washing twice for five minutes with the abovementioned DPBS-derived blocking buffer, cells were incubated with the secondary antibody in DPBS for 60 minutes at RT. After three washing steps for 10 minutes each with the same washing buffer, the nuclei were counterstained with 4′,6-diamidino-2-phenylindole (DAPI) (Roth, Cat. No. 6335.1, Karlsruhe, Baden-Württemberg, Germany) for 15 minutes at RT. Finally, coverslips were mounted onto microscope slides with the aqueous mounting medium Fluoromount^TM^ (Sigma Aldrich, Cat. No. F4680-25ML, MilliporeSigma, St. Louis, MI, USA).

### 2.4. Immunofluorescence Staining

The protocol described in this peer-reviewed article is published on protocols.io (dx.doi.org/10.17504/protocols.io.6qpvr84zolmk/v1, accessed on 11 September 2025) and is included for printing as a supporting information file with this article.

### 2.5. Tissue Processing After Immunofluorescence Staining Completion

#### 2.5.1. Dehydration and Paraffin Embedment

Tissue processing was carried out with the carousel-type tissue processor Citadel^TM^ 2000 Wax Bath (Thermo Fischer Scientific, Cat. No. 69810051, Shandon Scientific Ltd., Astmoor, England, UK) under reduced light exposure. 

Tissue specimens were placed into a high-density acetal polymer embedding cassette (e.g., Histosette^®^, Simport, Cat. No. M491-5, Bécancour, QC, Canada). For particularly thin samples, the cassettes were stuffed with biopsy sponge pads (e.g., NewcomerSupply, Cat. No. # 5106A, Middleton, WI, USA) to prevent sample loss during processing.

The cassettes were then placed into tissue baskets, then loaded onto the starting unit using hanger clips, according to the manufacturer’s instructions.

At our facility, the standard operating protocol (SOP) for millimeter-sized specimens is as shown in Table 1.

At the end of the abovementioned program, paraffin-embedded tissue blocks were prepared using a paraffin embedding tissue station (MEDITE, TES 99 600, Art. No. 46-0088-01, 02-9942-00, 02-9925-00, 02-9960-00, Herford, North Rhine-Westphalia, Germany) and allowed to solidify overnight at RT in the dark.

#### 2.5.2. Tissue Slicing

The paraffin blocks were initially stored at 4 °C, although they were placed at −20 °C overnight prior to slicing. Immediately after sectioning blocks at 3 µm thickness on a sliding microtome (Leica, Art. No. SM2010 R, Wetzlar, Hessen, Germany), sections floated on a 40 °C water bath containing distilled water. Subsequently, slices were transferred onto SuperFrost^®^ Plus adhesive microscope slides (Menzel/Epredia, Art. No. J1800AMNZ, Portsmouth, NH, USA) and left to dry overnight on a laboratory hot plate at 37 °C. Eventually, slides were stored at 4 °C and protected from light sources. 

#### 2.5.3. Deparaffinization, Rehydration and Mounting

Deparaffinization and rehydration were performed by immersing the slides sequentially in the following solutions: ready-to-use ROTI^®^Histol (Carl Roth, Art. No. 6640.1, Karlsruhe, Baden-Württemberg, Germany), three changes 5 minutes each; 100%, 96%, 80%, and 70% ethanol, 5 minutes each; and deionized water for 5 minutes.

Since tissue slices had already been stained, coverslips were directly mounted on the tissue samples using the aqueous mounting medium Fluoromount^TM^. Slides were dried overnight at RT, protected from light, and stored at 4 °C in the dark until imaging.

### 2.6. Imaging

#### 2.6.1. Three-Dimensional Imaging

Fluorescence images of the freshly stained tissue specimens were obtained by immunofluorescence microscopy using Zeiss Axio Examiner.Z1 with LSM 980 and Airyscan 2 and Zeiss ZEN 3.4 software (Zeiss, Jena, Germany). The technical specifications of the imaging system are listed in Table 2.

#### 2.6.2. Two-Dimensional Imaging

Fluorescence images of the abovementioned tissue slices were obtained by immunofluorescence microscopy using Zeiss Axio Observer (20×) and Zeiss ZEN 3.4 software (Zeiss, Jena, Germany).

### 2.7. Image Processing and Analysis

Acquired fluorescence images were processed using the software ImageJ (version 1.54f for Windows) [16]. Raw data obtained from 3D imaging were further rendered into 3D videos using Imaris (version 9.9.1; ©Oxford Instruments 2024), which can be found as Supplementary Materials.

For both of the abovementioned acquisition techniques, immunofluorescence staining was graded as positive or negative based on the presence or absence of immunofluorescence signals. Each GD2-immunostained sample was compared with its corresponding isotype control. Evaluation of the GD2 signal was performed independently by three investigators.

## 3. Results

### 3.1. Results from Antibody Validation 

**Figure 1:**
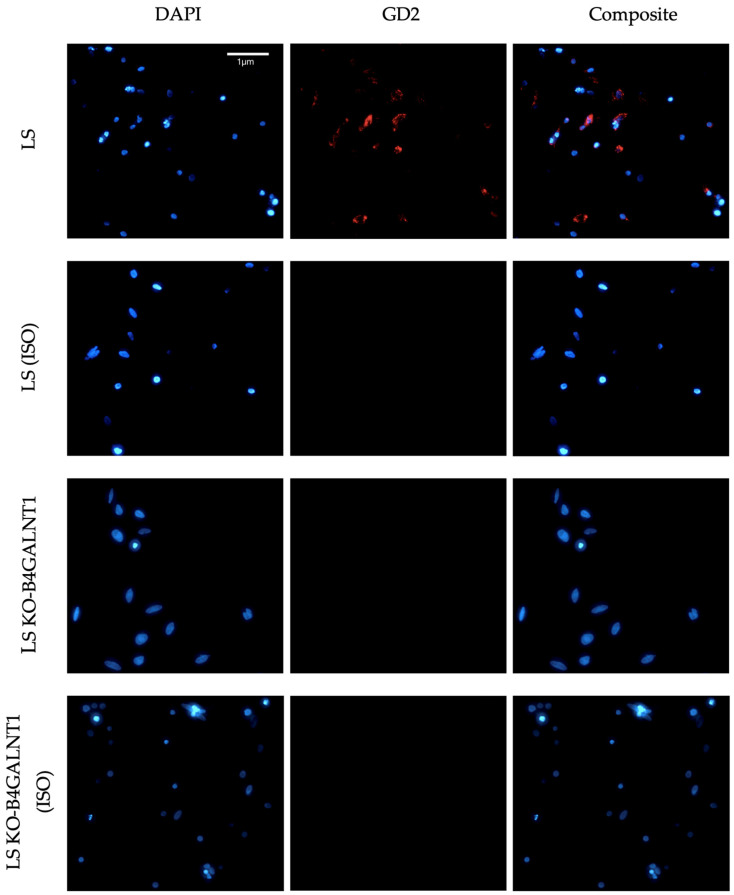
Fluorescence microscopy of the NB cell line LS for initial validation of the anti-GD2 antibody. LS and LS KO-B4GALNT1 cells were stained with the anti-GD2 antibody or an isotype control (ISO), followed by an Alexa Fluor^®^ 647 conjugated secondary antibody (red). Nuclei were counterstained with 4′,6-diamidino-2-phenylindole (DAPI, blue). Images were acquired using a Zeiss Axio Observer microscope (20×) and processed with the Zeiss ZEN 3.4 software. Representative images from each condition are shown.

In a preliminary phase of this work, the specificity of the GD2 antibody was tested on the NB LS cells and their GD2-deficient counterparts, LS KO-B4GALNT1 cells, as previously described. GD2 expression was confirmed in LS cells stained with Purified Mouse anti-Human Disialoganglioside GD2-Clone 14.G2a (BD Pharmingen™, Cat. No. 554272) as the primary antibody, whereas no signal was observed in the KO cells lacking GD2. Both cell lines were also stained with Purified Mouse IgG2a, κ Isotype Control (BD Pharmingen™, Cat. No. 556651), which produced no detectable signal (Figure 1).

### 3.2. Results from Protocol Establishment and Validation

The described protocol was developed stepwise by testing different concentrations and incubation times both for primary and secondary antibodies, as well as for DAPI, until optimal tissue staining conditions were assessed. To exclude cross-reactivity and non-specific antibody binding, specimens were stained with either only the selected primary or secondary antibody alone. In both cases, no signal was detected. Additionally, each sample was tested for possible tissue autofluorescence by substituting both primary and secondary antibodies with deionized water. Under these conditions, no signal was observed in any of the neuroblastoma, lymphoma and nephroblastoma samples.

**Figure 2:**
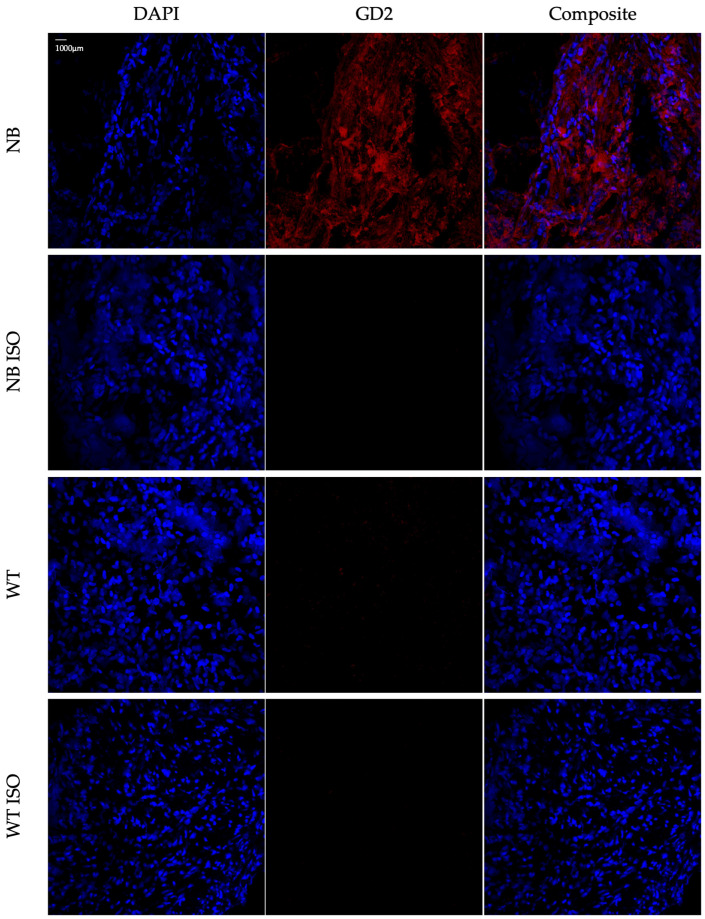
Representative GD2 immunofluorescence 3D imaging directly upon staining. Unfixed neuroblastoma (NB) and nephroblastoma/Wilms tumor (WT) samples were stained with either an anti-GD2 antibody or an isotype control (ISO), followed by an Alexa Fluor^®^ 647 conjugated secondary antibody (red). Nuclei were stained with DAPI (blue). Imaging was assessed after staining with the microscope Zeiss Axio Examiner.Z1 with LSM 980 (20×) and Airyscan 2.

Within a systematic approach to protocol development and validation, each of these preliminary tests were independently repeated at least three times, gaining consistent results. After establishing the novel staining protocol, it was applied to five neuroblastoma and five negative control samples, thereby assessing its reliability. Initial 3D analyses showed GD2 expression in all NB samples stained with the specific anti-human GD2 antibody. NB samples and negative controls stained with the isotype control antibody displayed no signal, except for occasional minimal unspecific background. Likewise, negative control specimens stained with anti-human GD2 antibody displayed none or only very weak signal (Figure 2; Supplementary Materials). These findings were completely consistent with available data regarding GD2 expression in bone marrow samples from the same patients, thereby validating the hereby presented protocol as specific and consistent.

**Figure 3:**
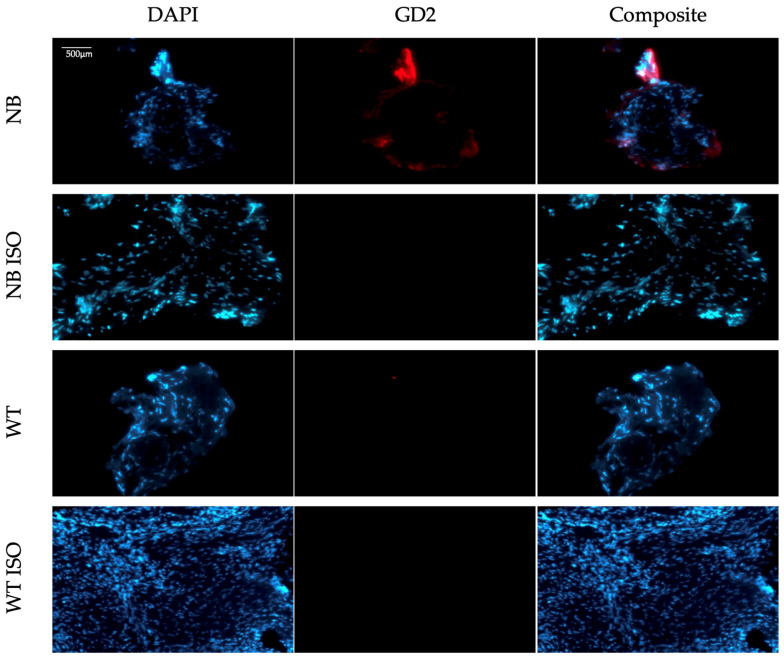
GD2 detection in 2D imaging of tumor samples following complete specimen processing. The stained samples underwent formalin fixation, dehydration and paraffin embedding. Resulting PPFE slices were deparaffinized, rehydrated and mounted. Images were acquired using the microscope Zeiss Axio Observer (20×). Shown are representative images of all samples. GD2 signal (red) and DAPI (blue) as nuclei counterstaining.

Three-dimensional immunofluorescence microscopy provides a detailed and spatially comprehensive view of the sample, which can significantly enhance the understanding of tissue architecture and marker localization. Since this technique might be challenging to access, we aimed to test the maintenance of the GD2 signal in slices from stained 3D tissue specimens. For this purpose, all stained specimens were fixed and further processed until FFPE slices were obtained, as previously described. GD2 expression remained detectable during subsequent 2D immunofluorescence microscopy of FFPE slices from positive controls stained with the anti-human GD2 primary antibody. Compared to the initial 3D imaging, however, the signal appeared weaker and was primarily localized in the peripheral areas of the tissue (Figure 3). This phenomenon might be due to either signal loss during further tissue processing after staining or differential detection capacity of the two IF microscopes. Importantly, the consistent retention of GD2 signal across all tested FFPE slices supports a broader and easier applicability of the developed staining protocol. In order to ensure broad applicability of the method presented herein, efforts aimed at the optimization of 2D imaging analyses may be undertaken. Therefore, while 3D microscopy represents a powerful research tool and may be used when available, our goal is to further refine the protocol in a way that maintains diagnostic utility, even when only 2D imaging is feasible.

**Figure 4:**
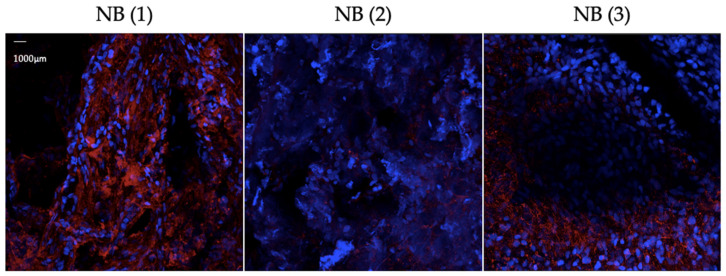
Heterogeneity of GD2 expression and distribution in different NB samples. Shown are representative images of three unfixed NB samples. GD2 signal (red) and DAPI (blue) as nuclei counterstaining. Imaging was assessed after staining with the microscope Zeiss Axio Examiner.Z1 with LSM 980 (20×) and Airyscan 2. NB (1) displays high intensity of GD2 signal, compared to NB (2). In both NB (1) and NB (2), the differential signal is homogenously distributed throughout the sample, unlike in NB (3).

Although the number of samples included in this work was limited (five NB and five negative control specimens), the results above support the feasibility of the protocol. Furthermore, we were able to observe noticeable differences in both the intensity and spatial distribution of GD2 staining across the analyzed 3D sample set (Figure 4). These variations are consistent with the known intra- and intertumoral heterogeneity of GD2 expression and highlight the importance of assessing not only the overall presence of GD2, but also its localization pattern within the tumor mass.

## 4. Discussion

Immunostaining is still a standard tool to render subcellular elements in fixed biological samples, including both cultured cells and tissue specimens. However, epitope recognition may vary depending on the fixation and paraffin embedding process as well as on the subsequent antigen retrieval steps. These steps can be avoided by performing IF on cryosections of frozen unprocessed tissue specimens. Nevertheless, frozen sections typically show poorer morphological preservation compared to FFPE ones and require a more careful storage [17,18].

Most GD2 staining techniques are briefly described in the Materials and Methods section of broader framework studies and often lack detailed descriptions of each step, which would foster reproducibility and allow a more sustainable research approach, as we aimed with this article [19,20]. Thus, in this study we aimed to develop and accurately describe a protocol for the detection of GD2 directly in primary NB tumor tissue. 

A possible challenge to overcome, while applying our protocol, is the availability of IF microscopes. Standard light microscopes are more widespread, as they are easier to afford and handle; however, they are restricted to the analysis of immunohistochemically (IHC)-stained samples. Although IHC represents a capstone in histopathology and research, it is marked by several limitations, such as difficult simultaneous study of multiple antigens, semi-quantitative interpretation with high intra- and interobserver variability, insufficient protocol standardization, and inconsistent reproducibility of results [21]. Since diagnostic, therapy and follow-up of neuroblastoma patients is usually performed in reference centers, we presume and claim the presence of research units and pathology departments with open access to all the required facilities, i.e., IF microscopy platforms.

In some tissue specimens, cytolysis and compromised nuclear morphology could be observed, which may be own to osmotic cellular damage due to the lack of prior tissue fixation. However, since these effects were limited only to some specimens, intrinsic tumor characteristics may influence tissue stability throughout the staining process. To improve structural preservation and signal consistency in FFPE slices, an optimization of the hereby presented protocol may be pursued, thereby facilitating precise GD2 signal localization and ameliorated cell segmentation. These, in turn, are key aspects for a proper tissue analysis as shown by precedent GD2 staining protocols of fresh frozen sections [9], and also for other tumor entities expressing this surface marker. In such cases, GD2 signal occasionally displays a turbid fluorescent pattern, thereby compromising the reliability and consistency of the results [22]. Further attempts to stain GD2 in fixed tissue sections using immunohistochemistry have revealed unexpected GD2 presence and distribution. As in the work of Zhong and colleagues, off-target staining was observed, although GD2 expression was positive in 35% of the analyzed breast carcinoma samples. Moreover, in neoplastic areas, GD2 signal was confined to the perinuclear and cytoplasmic regions, rather than the characteristic cell membrane localization, thus limiting the specificity of this method [23].

An alternative approach for GD2 detection in diagnostic workflows for NB is flow cytometry (FC). Although FC enables semi-quantitative analysis of GD2 signals, this method requires samples to be in single-cell suspension, necessitating prior manipulation of the tissue (i.e., cell isolation, culture, and passaging), which can alter GD2 expression, as already observed with other markers. Additionally, FC provides no information on signal distribution, thereby reducing information about GD2 expression patterns [24].

Furthermore, molecular and proteomic assays are not suitable due to GD2’s non-protein nature. Indirect assessment of GD2 expression via the presence of B4GALNT1 is not feasible either, as the enzyme’s expression does not directly correlate with GD2 surface levels [24]. Similarly, attempts to identify a gene expression signature for specific oncotypes highlighted B4GALNT1 and ST8SIA1 (a ganglioside synthesis enzyme—GD3 synthase) as a potential binary predictor of a GD2-positive phenotype. However, the limited and heterogeneous data volume renders these findings preliminary and not yet reliable for diagnostic application [25].

To date, GD2 immunostaining remains the method of choice. A recent study demonstrated successful GD2 staining using IF on FFPE specimens. In this work, protocol specificity was validated by staining patient-derived cells previously confirmed as GD2-negative via FC. Despite promising results, the authors reported several technical limitations, including issues related to antigen retrieval, blocking buffer composition, and signal detection. If not carefully optimized, these steps lead to background staining and loss of specificity [26].

In contrast, the protocol presented here offers several advantages, such as eliminating the need for antigen retrieval and reducing both primary antibody incubation time and concentration. In addition, it allows the characterization of (1) the degree of GD2 expression (in a semi-quantitative manner) and (2) the accurate spatial localization of GD2. As already underlined by other authors [27], primary neuroblastoma tumor samples constitute a group of heterogeneous-expressing GD2-positive cells, making it insufficient to simply assess the presence or absence of GD2 expression. However, this important aspect remains neglected when it comes to applied therapeutic approaches. For this reason, the optimization of the hereby presented protocol may hold potential as a future diagnostic tool.

## 5. Conclusions

We successfully established and validated a novel protocol for reliably performing immunostaining of the membrane antigen GD2 in unfixed neuroblastoma tissue samples, enabling subsequent fluorescence microscopy, imaging and analysis. Despite a few limitations that are already outlined, this staining workflow enables relatively rapid assessment of GD2 positivity or negativity directly in primary tumor samples and may thus represent a relevant diagnostic tool within the context of tumor staging and precision medicine.

Moreover, this protocol may be applied for the staining of other surface markers, such as GD3 or GM2, thereby facilitating a more individualized NB patient profiling and, therefore, therapeutic assessment.

## Figures and Tables

**Table 1:** SOP for dehydration and paraffin embedment. Description of the twelve-step tissue dehydration and paraffin infiltration processing program used prior to FFPE block preparation.

Reagent	Step Duration
70% isopropanol	50 min
70% isopropanol	60 min
80% isopropanol	60 min
85% isopropanol	60 min
90% isopropanol	60 min
90% isopropanol	60 min
96% isopropanol	60 min
96% isopropanol	60 min
100% isopropanol ^1^	90 min
100% isopropanol	90 min
Wax bath with paraffin ^2^ (56 °C)	120 min
Wax bath with paraffin ^2^ (56 °C)	180 min

^1^ Isopropanol (Chemsolute^®^, Art. No. 1136.5000, TH Geyer, Böblingen, Baden-Württemberg, Germany). ^2^ Tissue-Tek^®^ III paraffin wax (Sakura, Cat. No. 4511, Torrance, CA, USA).

**Table 2:** Three-dimensional imaging system. Technical description of the components used during immunofluorescence microscopy with Zeiss Axio Examiner.Z1 with LSM 980 and Airyscan 2 [15].

Objective	20× W Plan-APOCHROMAT Corr DIC Refractive index: 1.33–1.36 Numerical Aperture: 1.0 Working Distance (mm): 2.4		
Detectors	Confocal unit: −32× GaAsP −2× PMT (UV and NIR) −Airyscan 2		
Laser lines (nm)	SpectraPhysics InSight X3+	680–1030 (tunable)	1040 (fixed)
Filters for fluorescence	For 32+2 channel spectral detection: tunable between 380 and 900 nm For eyepiece: Filter system (em.-color, dye): excitation | beamsplitter | emission • 49 (blue; AF405, DAPI): BP 365 | FT 395 | BP 445/50 • 38 (green; AF488, GFP): BP 470/40 | FT 495 | BP 525/50 • 43 (red; AF568, mCherry): BP 550/25 | FT 570 | BP 605/70 For Airyscan: 1. BP 420-480 + BP 495-550 2. BP 420-480 + BP 570-630 3. BP 420-500 + LP 605 4. BP 465-505 + BP 525-585 5. BP 495-550 + BP 570-630 6. BP 495-560 + LP 660 7. BP 570-620 + LP 660		
UV-VIS illumination	HXP 120 V		
Halogen lamp	100 W 12 V		
Actively damped optical table	Newport Smart Table UT2		

## Data Availability

The data that support the findings of this study are available from the corresponding author upon request.

## Supplementary Materials

The following supporting information can be downloaded at https://www.mdpi.com/article/10.3390/cells14181462/s1, Video S1: GD2 IF—NB sample; Video S2: GD2 IF—NB sample (ISO); Video S3: GD2 IF—WT sample; Video S4: GD2 IF—WT sample (ISO).

## Abbreviations

NB	Neuroblastoma
mAb	Monoclonal antibody
CAR	Chimeric antigen receptor-expressing
FFPE	Formalin-fixed paraffin-embedded
IF	Immunofluorescence
AF	Alexa Fluor
KO	Knock out
RT	Room temperature
FBS	Fetal bovine serum
(D)PBS	(Dulbecco’s) phosphate-buffered saline
BSA	Bovine serum albumin
DAPI	4′,6-diamidino-2-phenylindole
SOP	Standard operating protocol
WT	Wilms tumor
GFP	Green fluorescent protein
IHC	Immunohistochemistry
FC	Flow cytometry
